# Urban spatial dynamic modeling in transition: Hybrid, human-centric, and scalable futures

**DOI:** 10.1007/s13280-026-02392-0

**Published:** 2026-05-14

**Authors:** Zipan Cai

**Affiliations:** 1 Department of Urban Planning, School of Architecture, Southeast University, Sipailou 2, Nanjing, 210096 China; 2 Department of Sustainable Development, Environmental Science and Engineering (SEED), KTH Royal Institute of Technology, Teknikringen 10B, 100 44 Stockholm, Sweden

**Keywords:** Foundation models, Graph theory, Planning support systems, Space syntax, Spatial dynamic modeling, Sustainability transitions

## Abstract

Urban spatial dynamic modeling (SDM) has long been used to anticipate land-use change, urban expansion, and infrastructure demand. Yet, despite increasing methodological sophistication, most applications remain oriented toward reproducing spatial patterns rather than supporting sustainability transitions. This perspective argues that SDM is at a turning point and outlines how it can evolve to address climate neutrality, resilience, and equity in both growing and shrinking cities. Five interlinked development pathways are proposed: (i) hybrid AI–theory models balancing predictive accuracy and interpretability; (ii) human-centric accessibility frameworks grounded in time geography; (iii) network-based resilience approaches drawing on graph theory and space syntax; (iv) semantic place functions enabled by vision–language foundation models; and (v) scalable architectures applicable across diverse data and governance contexts. Together, these pathways reposition SDM from a forecasting tool to a transition-support instrument, aligning it with planning support systems and enabling more sustainable and just urban futures.

## Introduction

Cities are widely recognized as pivotal arenas for addressing global sustainability challenges. More than half of the world’s population already lives in urban areas, a share expected to rise to nearly 70% by 2050 (IPCC 2023). Cities generate over 70% of global CO_2_ emissions and consume the majority of the world’s energy (UN-Habitat 2020). At the same time, they are on the front lines of climate-related risks, facing heightened exposure to flooding, heatwaves, and pollution (IEA 2021). Urban areas are also essential sites for implementing solutions, where interventions in infrastructure, land use, and governance can deliver rapid and large-scale benefits (Evans et al. 2016). Anticipating urban change has therefore become an essential task for researchers and policymakers, shaping pathways toward resilient, low-carbon, and equitable futures (Meerow and Newell 2019).

While many global analyses emphasize spatial expansion, a substantial share of 60–70% contemporary urbanization arises from internal population growth, densification, and administrative reclassification from rural to urban areas (Anestis and Stathakis 2024). This “silent urbanization” is particularly prevalent in the Global South, where rapidly transforming localities often lack the fiscal and technical capacity to match rising demands for housing, infrastructure, and services (Schindler 2017). In many of these regions, silent urbanization unfolds in informal or unplanned settlements that encroach on flood-prone lands or steep slopes, exacerbating exposure to climate hazards (Williams et al. 2019). In the absence of commensurate formal planning and investment, such communities face chronic deficits in housing, sanitation, and transportation networks (McGranahan 2015).

Addressing these challenges calls for new approaches: In particular, co-production processes, defined as collaborative efforts in which local communities, authorities, and researchers jointly develop knowledge, scenarios, and implementation options, can be transformational in unplanned, hazard-prone urban areas (Bixler et al. 2022). Co-production leverages local knowledge and community ownership to upgrade basic services, reduce risk, and bridge capacity gaps in informal settlements (Johnson et al. 2022). Foundational studies have mapped the scale and consequences of projected urban expansion (Seto et al. 2012), while more recent contributions highlight how urban systems themselves can act as leverage points for sustainability transformation (Bai 2025). This aligns with calls for a “new urban science” advancing interdisciplinary and transdisciplinary approaches to sustainable urban transformations (Karvonen et al. 2021).

In this perspective, we introduce the term spatial dynamic modeling (SDM) as an umbrella concept to describe the diverse family of approaches that explicitly simulate urban spatial change (Cai 2025). By using this term, we emphasize the spatially explicit and dynamic character of these models, situating them within the broader landscape of planning support science. Early traditions such as cellular automata (CA) demonstrated how simple neighborhood rules could generate realistic urban patterns (White and Engelen 1993), while agent-based models (ABMs) foregrounded the heterogeneous behavior of households, firms, and planners (Parker et al. 2003). Subsequent developments combined statistical, hybrid, and machine learning methods to capture more complex dynamics (Clarke et al. 1997; Li and Gong 2016). We have previously extended this tradition by applying SDM for scenario planning and for analyzing amenity-driven urban change (Cai et al. 2020, 2023). At the same time, broader perspectives from scaling theory and urban science offer theoretical grounding, showing how cities can be understood as complex, self-organizing systems (Bettencourt 2007, 2013).

Recognizing the diverse trajectories of contemporary urbanization reinforces the need for SDM to evolve beyond forecasting patterns of growth alone. We further position SDM within a social–ecological perspective that links resilience thinking and the Anthropocene framing to debates on urban sustainability transitions (Steffen et al. 2011; Pickett et al. 2024). This perspective highlights not only the adaptive capacities of urban systems but also their embeddedness in broader social and ecological dynamics. Analogies from resilience and governance research illustrate the governance challenges along expanding urban frontiers (Beilin and Wilkinson 2015). Insights from resilience and related governance literatures underscore that institutional design and community engagement shape conflict and cooperation in rapidly transforming urban landscapes (Sharifi and Yamagata 2018). Though drawn from ecological contexts, these insights also illuminate urban institutional challenges.

Despite these conceptual advances, mainstream SDM practice remains constrained. Too often, it prioritizes pattern reproduction over purposeful transitions, depends on data-intensive calibration with limited transferability, assumes perpetual growth in contexts of shrinkage, and produces outputs poorly aligned with policy levers. Moreover, resilience and systemic shocks remain underexplored. These limitations, elaborated in “Limitations of current SDM practice” section, reveal why SDM’s contributions to sustainability transitions have so far been modest relative to its technical sophistication.

In this perspective, we argue that SDM now stands at a turning point. Rather than being judged solely by how well it replicates past patterns, its value must be assessed by how effectively it helps cities navigate uncertain pathways toward climate neutrality, resilience, and equity. We outline five interlinked pathways for repositioning SDM: (i) hybrid AI-theory models that balance interpretability and predictive performance; (ii) human-centric accessibility frameworks grounded in time geography, particularly the space–time prism; (iii) network-based resilience approaches drawing on graph theory, multilayer networks, and space syntax; (iv) semantic place functions enabled by foundation models such as vision–language architectures; and (v) scalable and transferable frameworks that make SDM usable for cities of all sizes and trajectories. These pathways redefine the role of SDM, shifting it from a forecasting exercise toward a transition-support platform, and positioning it within the broader tradition of planning support systems (PSS) to address urgent policy agendas.

## Limitations of current SDM practice

Urban SDM has become one of the most widely applied approaches for simulating and anticipating urban land-use change. Since the 1990s, CA and agent-based frameworks have been deployed to reproduce urban expansion, forecast infrastructure demand, and evaluate alternative growth scenarios. The methodological sophistication of the field is impressive, and its scientific contributions significant. Nevertheless, SDM as it is commonly practiced today faces a series of structural limitations that constrain its usefulness for the pressing challenges of climate neutrality, resilience, and equity. These limitations are not marginal; they shape how models are designed, calibrated, and interpreted.

### Pattern reproduction over purposeful transitions

A defining characteristic of most SDMs is their emphasis on reproducing spatial patterns. Model performance is typically judged by comparing simulated maps against observed historical land-use change, using measures such as kappa statistics or figure of merit (Cai et al. 2023). This emphasis on technical accuracy has undoubtedly spurred methodological innovation in calibration procedures, validation techniques, and spatial metrics. Yet it has also narrowed the scope of inquiry. As Filatova et al. (2013) observed in their review of ABM, success is too often defined as the ability to replicate what has already occurred, rather than the ability to illuminate strategies for what ought to happen next.

This retrospective orientation has had important consequences. SDM has excelled at answering questions such as “where growth is likely to occur,” but has been far less frequently applied to questions like “which interventions reduce vulnerability to floods?” or “how might a proposed transport policy improve equitable access?” By focusing so heavily on spatial fit, the field has become methodologically rigorous but strategically shallow. For SDM to remain relevant, its evaluative lens must expand beyond accuracy metrics to encompass outcomes such as accessibility, resilience, and justice.

### Data intensity and uneven applicability

Another persistent limitation lies in the heavy data requirements of most SDM frameworks. Reliable calibration often depends on multi-decadal land-use records, detailed socioeconomic datasets, and finely resolved accessibility layers. While these resources exist in a handful of large, well-documented metropolitan regions, they are often absent in smaller or resource-constrained cities. Wu and Silva (2010) described this as a persistent “implementation gap,” whereby the most sophisticated models are least likely to be adopted precisely where they are most needed.

The reliance on extensive calibration also undermines transferability. A model finely tuned to the dynamics of Shenzhen or Los Angeles may fail entirely when applied in Nairobi or Stockholm. This uneven geography of modeling capacity mirrors broader global inequalities in urban research: The Global North dominates case studies, while many of the fast-growing cities in the Global South remain underrepresented (Zhou et al. 2022). This imbalance is not only a scientific problem but also an ethical one, since it risks excluding the very cities undergoing the steepest transitions.

Uncertainties in baseline urban land datasets further complicate the picture. As Chakraborty et al. (2024) have recently demonstrated, estimates of urban land can vary dramatically across scales and sources, creating divergent inputs for calibration. Without addressing these uncertainties, SDM risks building on shaky empirical foundations, undermining its ability to inform robust policy.

### Growth bias in a world of shrinking cities

Most SDMs are designed with an implicit assumption of perpetual growth. Their primary task is to allocate new development across space, reflecting the dominant concern of rapid urbanization in the late twentieth century. Yet today, a growing number of cities are shrinking. Between 1990 and 2010, more than a quarter of European cities experienced population decline (Haase et al. 2016), with similar trajectories observed in Japan and North America (Martínez-Fernández et al. 2012).

Shrinking cities confront distinct challenges, such as decommissioning surplus housing and infrastructure, consolidating public services, and maintaining accessibility for dispersed populations (Gao et al. 2023). Traditional SDM frameworks that assume expansion are poorly suited to these contexts and may even encourage maladaptive strategies by reinforcing growth-oriented planning paradigms. Despite the growing recognition of shrinkage as a critical issue, explicit shrinkage-aware SDM applications remain rare. This represents a decisive gap, since strategies such as selective densification, network rightsizing, and service preservation demand tailored modeling support.

### Disconnect between outputs and policy levers

Even when SDMs produce technically impressive forecasts, their outputs often fail to connect with the realities of governance. A probability surface indicating where sprawl is likely to occur does not map neatly onto the instruments available to planners, transport agencies, or housing authorities. Voinov and Bousquet (2010) emphasize that models gain legitimacy in decision-making contexts only when they can illuminate specific choices and trade-offs. Yet most SDM outputs continue to be abstract, providing little guidance on which zoning reform, infrastructure investment, or land management strategy best advances resilience or equity.

This disconnect helps explain why, despite thousands of academic applications, SDM has seen limited institutional uptake in planning practice. The issue is not that planners are uninterested in modeling, but that models often fail to speak in actionable terms. Unless SDM aligns more closely with the decision spaces of governance, it risks remaining a primarily academic pursuit. A similar gap has been observed in the development of PSS, where technically sophisticated tools often struggled to gain traction in practice due to limited usability or misalignment with institutional routines (Geertman and Stillwell 2020).

**Fig. 1:**
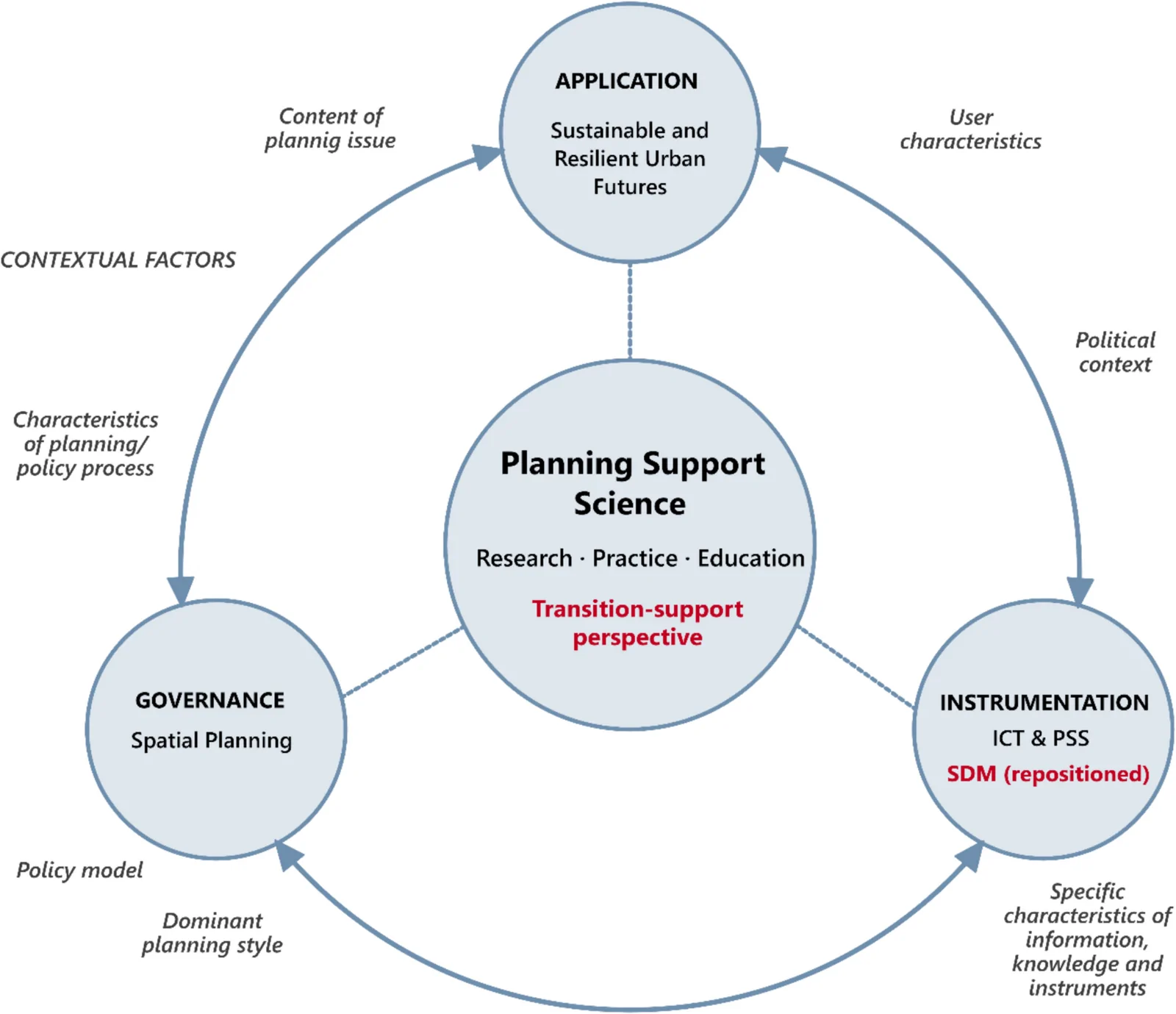
Positioning SDM within the planning support science framework (adapted from Geertman and Stillwell 2020). The diagram highlights SDM as part of the instrumentation domain, linking governance and application to support sustainable and resilient urban futures

As Geertman (2016) and colleagues argue, PSS encompasses instrumentation, governance, and application in support of sustainable urban futures. In this framing, SDM sits in the instrumentation domain but links directly to governance and application, clarifying why uptake depends as much on workflows and decision levers as on model accuracy (Fig. 1).

### Underdeveloped attention to resilience

SDM has yet to adequately engage with the realities of resilience. Contemporary cities are increasingly defined by shocks: floods, heatwaves, pandemics, financial crises, and infrastructure breakdowns. Yet most SDMs extrapolate land‐use change as if systems were stable, ignoring thresholds, tipping points, and cascading failures (Cai 2025). Urban systems are complex adaptive systems with resilience as a defining property (Boutreux et al. 2024). By neglecting resilience-oriented modeling, SDM scenarios risk producing smooth forecasts that obscure systemic vulnerabilities.

There are encouraging but isolated exceptions. Some recent studies have overlaid hazard maps onto SDM outputs to explore climate risk exposure (Liu et al. 2017), while others have linked land-use change to ecosystem service dynamics (Verburg et al. 2019). However, these examples remain peripheral rather than mainstream practice. If SDM is to contribute meaningfully to sustainability transitions, resilience must move from the margins to the center of model design and evaluation.

### Toward a transition in SDM

Taken together, these limitations explain why SDM, despite its technical sophistication, often struggles to influence planning for sustainability transitions. The emphasis on reproducing patterns rather than testing strategies, the reliance on data-rich contexts, the assumption of perpetual growth, the weak connection to policy levers, and the limited attention to resilience all constrain the field. These are not minor issues; they are systemic features that must be addressed if SDM is to remain relevant.

The path forward is not to discard existing methods but to reposition them. SDM needs to move from a retrospective orientation to a prospective one, from forecasting maps to illuminating strategies, from privileging data-rich megacities to supporting all types of cities, and from ignoring shocks to embedding resilience.

## Future pathways for an innovative and usable SDM

**Fig. 2:**
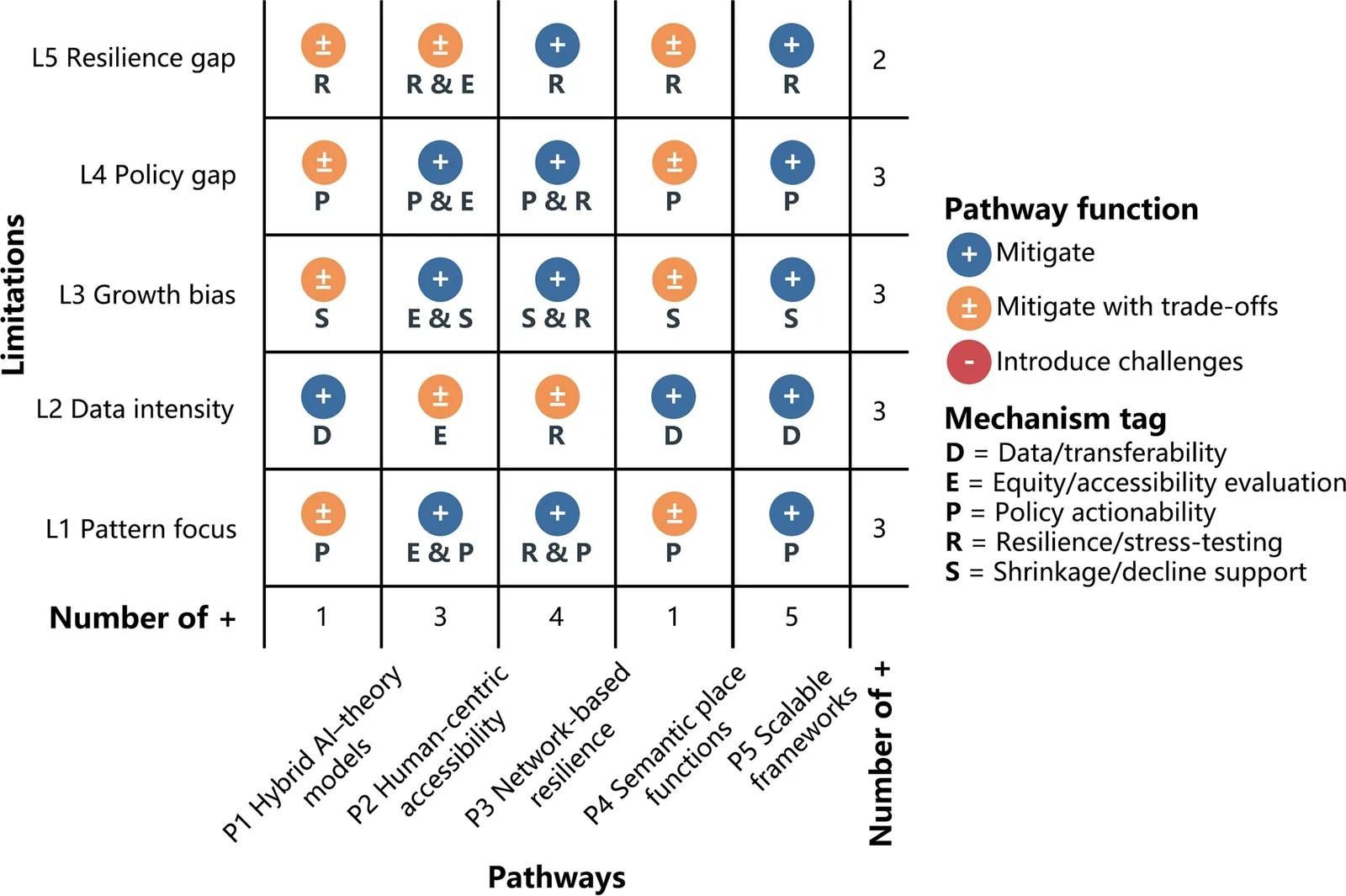
Conceptual mapping between five SDM pathways and key limitations of current SDM practice. Symbols indicate whether a pathway mitigates (+), mitigates with trade-offs (±), or introduces challenges (−), and mechanism tags denote the dominant mode of contribution

The limitations outlined in “Limitations of current SDM practice” section point to directions for change rather than a single technical fix. In response, we develop five pathways that together reorient SDM practice toward climate neutrality, resilience, and equity. Rather than defining a new monolithic model or proposing one-to-one solutions to individual limitations, these pathways represent complementary and interlinked shifts in how SDM is specified, evaluated, and implemented: combining theory-driven structures with machine learning, embedding time-geographic accessibility, integrating network-based resilience metrics, enriching suitability with semantic place functions, and designing scalable architectures that work across diverse urban contexts. Each pathway addresses multiple limitations through distinct mechanisms, and each limitation requires contributions from several pathways. Figure 2 summarizes these many-to-many linkages and shows whether each pathway mitigates, mitigates with trade-offs, or may introduce challenges; the following subsections elaborate each pathway in turn.

### Hybrid AI–theory models

A first pathway lies in combining the interpretability of traditional models with the predictive strength of artificial intelligence. CA and ABM continue to be valuable precisely because their rules are explicit and interpretable. Planners can understand why a neighborhood grows, or how actor decisions shape land-use patterns. Yet such models often underperform when dynamics are nonlinear, multiscalar, or poorly specified. By contrast, machine learning methods ranging from random forests and gradient boosting to deep neural networks excel at pattern detection in high-dimensional data (Liu et al. 2021). Applications in urban studies have shown impressive predictive performance from satellite imagery, mobility traces, and socioeconomic indicators (Liu et al. 2017).

The problem, as Rudin (2019) has argued more broadly, is that “black-box” models are difficult to interpret and hard to trust in governance contexts. Here, hybridization offers a way forward. Machine learning can be used to estimate suitability surfaces, behavioral parameters, or transition probabilities that feed into rule-based CA or ABM cores. In this setup, the model retains a transparent causal structure, while learning components improve predictive performance. Alternatively, hybrid approaches can constrain machine learning with domain knowledge, a principle reflected in theory-informed modeling traditions (Batty 2013). Advances in vision transformers (Dosovitskiy et al. 2021; Radford et al. 2021) and remote sensing vision-language models (Huang et al. 2024; Liu et al. 2024; Lu et al. 2025) further enable these strategies.

Hybrid SDM has several advantages. First, it can reduce calibration demands by allowing learned components to generalize across contexts, thereby improving transferability. Second, it balances performance with trust: Explicit rules continue to be open to scrutiny, while AI handles complexity. Third, hybridization supports participatory modeling. Stakeholders can engage with rule-based modules for debating assumptions about neighborhood influence or actor behavior without needing to understand every detail of the machine learning underpinnings.

Of course, risks remain. Hybrid models can still reproduce biases present in training data, and their validation requires careful design. But in our judgment, this is the most promising path to balance predictive accuracy with interpretability. Hybrid AI–theory models will not replace SDM as we know it; they will become SDM’s next evolutionary step.

### Human-centric accessibility

A second pathway is to embed human accessibility at the core of SDM evaluation. Since Hägerstrand’s seminal work, time geography has provided a formal framework for understanding the constraints that shape daily life. A central operational concept is the space–time prism, which represents the feasible set of activities an individual can undertake given fixed anchors, travel speeds, and time budgets (Hägerstrand 1968). This concept operationalizes time geography into a measurable construct that can be integrated directly into SDM, allowing accessibility to be evaluated dynamically across scenarios of land-use and transport change. Over the past two decades, the prism idea has been extended to multimodal transport, congestion, and even affordability, making it increasingly relevant for policy evaluation (Geurs and van Wee 2004; Neutens et al. 2011).

Integrating these ideas into SDM would transform how futures are assessed. A new metro line, for instance, could be evaluated not just for induced development patterns but for how it enlarges prisms for low-income groups or caregivers constrained by rigid schedules (Pereira et al. 2017). Land-use densification strategies could be assessed for whether they reduce inequities in opportunity access, as well as for whether they shift land-use probability surfaces. Accessibility reframes the criteria of success: Urban models should be judged not only by how accurately they reproduce past spatial patterns but also by how well they expand feasible opportunities for diverse groups. By embedding the space–time prism within SDM, it becomes possible to quantify these opportunity sets under alternative policies—making equity and inclusivity central evaluation dimensions alongside traditional accuracy.

Accessibility also matters profoundly for shrinking cities. When schools or clinics close, residents may face sharp reductions in feasible access, even if land-use change probabilities appear stable. An SDM that incorporates space–time constraints could identify which facilities are most critical to preserve, guiding selective investment in the face of demographic decline (Cai et al. 2026). In this way, accessibility provides a common language that connects modeling to governance, bridging the gap between technical outputs and lived realities.

We argue that repositioning SDM around accessibility is not simply a methodological refinement. It is a normative commitment to equity. By embedding time-geographic principles, SDM becomes a tool for evaluating fairness in urban futures.

### Network-based resilience

A third pathway centers on the networks that underpin urban life. Cities operate through interconnected systems of transport, utilities, green–blue infrastructures, and digital flows. These systems both enable daily activity and create vulnerabilities. Network disruptions, from floods that disable metro lines to power outages that cascade through water supply, can paralyze urban functions. Yet conventional SDMs often treat networks as static suitability factors, not as dynamic systems with their own fragilities.

Network science provides powerful methods for addressing this gap. Metrics and models that capture robustness, interdependence, and cascading failure are now well established (Kivelá et al. 2014; De Domenico et al. 2016; Gao et al. 2016). Embedding these within SDM would enable futures to be stress-tested: A proposed growth corridor could be evaluated for how robust its accessibility continues to be if a flood disables a key bridge.

Equally pivotal is the tradition of space syntax, which analyzes the spatial configuration of urban form. Measures such as integration and choice have been shown to correlate with pedestrian flows, retail vitality, and social interaction (Hillier and Hanson 1989; Penn 2003; Peponis et al. 2008). Incorporating space syntax into SDM could reveal how redesigning street networks through superblocks, pedestrianization, or green corridors, which can further reshape both accessibility and resilience.

For shrinking cities, network analysis is particularly essential. As populations decline and services are withdrawn, networks can “hollow out,” leaving residents increasingly disconnected (Carson et al. 2022). Resilience-oriented SDM could help planners identify decisive nodes and links to preserve, ensuring basic accessibility despite consolidation.

Resilience cannot continue to be peripheral to SDM. By integrating graph theory, multilayer networks, and space syntax, SDM can move from describing growth to evaluating robustness. This makes SDM not only predictive but also a planning partner for shocks that will shape urban futures.

### Semantic place functions

A fourth pathway focuses on enriching SDM with semantic layers that capture how urban spaces are actually used and experienced. Traditional SDMs rely heavily on land-use categories or points-of-interest datasets. While useful, these inputs are often coarse, outdated, or inconsistent across regions. They reduce cities to rigid categories that fail to capture everyday functions such as informal retail, cultural clusters, or shaded pedestrian corridors.

Recent advances in foundation models, particularly vision-language models (VLMs), provide a powerful alternative. CLIP, for example, was trained on hundreds of millions of image-text pairs, making it a prototypical foundation model that can recognize semantic categories with little or no supervision (Radford et al. 2021). Early applications in remote sensing and urban imagery show that VLMs can enrich suitability surfaces and support transferability across cities (Huang et al. 2024; Liu et al. 2024).

For SDM, the implications are significant. Semantic functions derived from VLMs can enrich suitability surfaces, support transferability across cities, and provide timely functional layers even in data-poor contexts. This lowers the barrier for participation, particularly for cities in the Global South where detailed land-use surveys may be unavailable. So far, such semantic enrichment has not yet been mainstreamed in SDM, but it represents a pivotal frontier.

VLMs inherit biases from their training data, and their classifications need to be validated carefully. Participatory verification, in which local stakeholders review and refine semantic outputs, could mitigate these risks. Thus, the potential benefits outweigh the risks: By moving beyond rigid land-use categories, SDM can represent cities in more meaningful human terms. This makes SDM more relevant for planning questions that hinge not only on where change occurs, but on what kind of place emerges.

### Scalable and transferable frameworks

The final pathway concerns the scalability of SDM. At present, sophisticated applications are concentrated in data-rich megacities, while smaller and resource-constrained cities continue to be largely excluded. This imbalance mirrors wider inequities in urban research and risks exacerbating divides in planning capacity (Wu and Silva 2010). The future of SDM needs to be inclusive: It ought to be usable by every city, regardless of size, growth trajectory, or data availability.

One promising approach is a two-tier architecture. At its core, a lightweight, transferable model could run with minimal data, drawing on open semantic priors and remote sensing. On top of this core, modular extensions could allow richer analysis where resources permit, for example, adding detailed agent-based simulations or sectoral couplings. This design ensures that every city can run a baseline SDM, while better-resourced cities can integrate additional complexity.

For shrinking cities, scalability also requires explicit modeling of decline. Shrinkage-aware SDMs could simulate selective densification, service consolidation, and infrastructure rightsizing, helping municipalities manage depopulation without sacrificing equity (Zhang and Cai 2026). At present, such capabilities are rare, leaving shrinking cities without adequate modeling tools.

Transferability also requires shared standards. Open data, model cards, and transparent evaluation metrics would make results comparable across contexts and reduce misinterpretation (Geertman 2006; Geertman and Stillwell 2020). International collaboration, combined with advances in urban analytics and digital infrastructures, could accelerate progress (Batty 2018).

To sum up, scalability is not simply a technical issue but an ethical one. The benefits of SDM should not be limited to wealthy megacities. By designing frameworks that are lightweight, modular, and shrinkage-aware, SDM can become a genuinely global tool, supporting municipalities across diverse geographies in their pursuit of sustainability transitions.

## Repositioning SDM for sustainability transitions

**Fig. 3:**
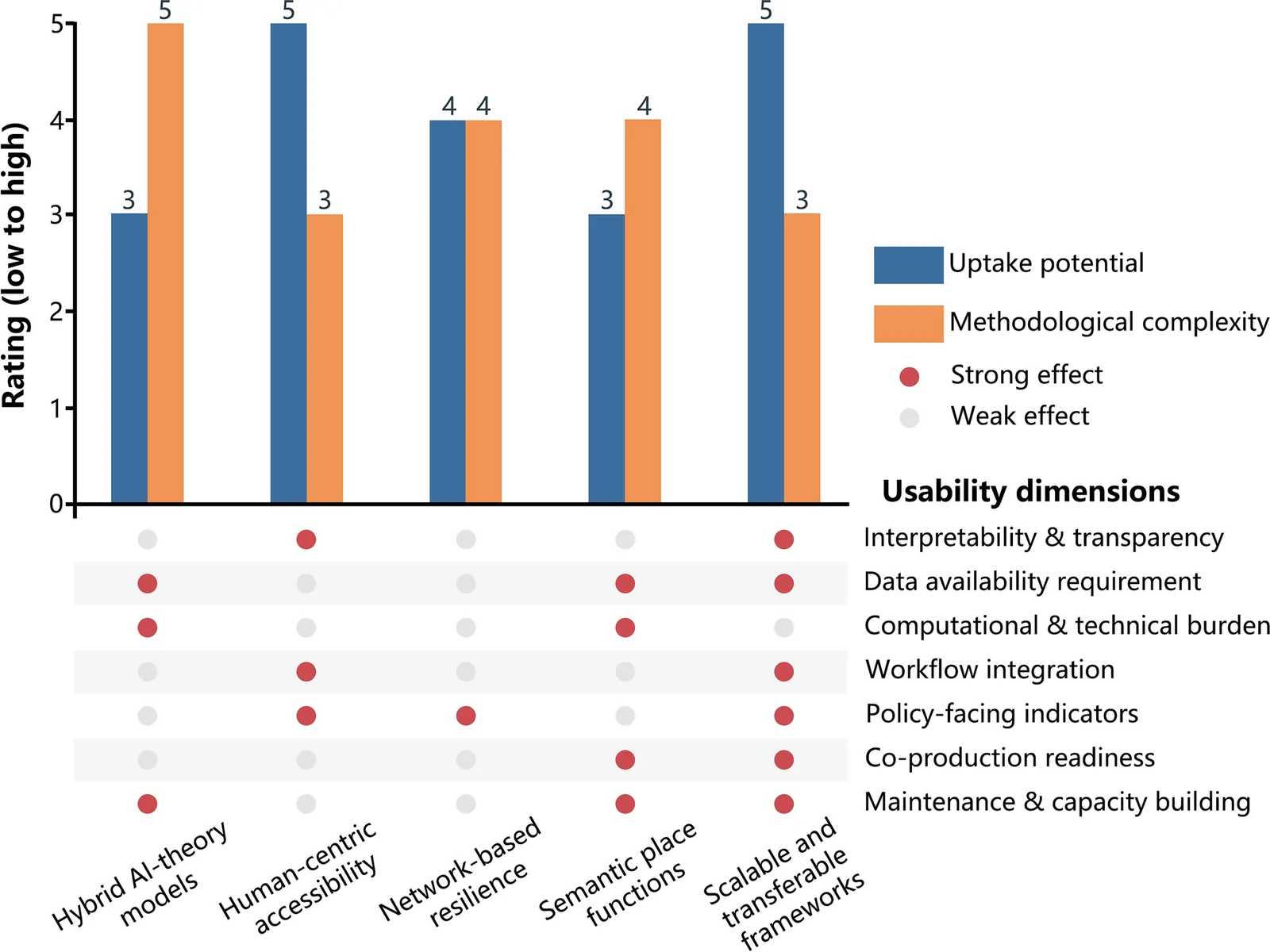
Usability and practical uptake dimensions of the five SDM pathways. Bar heights indicate uptake potential, with methodological complexity annotated for each pathway. The dot-matrix summarizes how each pathway interacts with key usability dimensions

A critical barrier to repositioning SDM for sustainability transitions is the tension between methodological sophistication and practical usability, which can limit uptake in real planning and policy contexts. Figure 3 summarizes this complexity–usability tension and shows how each pathway interacts with key usability dimensions; this synthesis motivates the research and policy agendas below, which focus on lowering adoption barriers through interpretability, workflow integration, co-production, and capacity building.

The pathways outlined above are not isolated innovations. They are complementary shifts that together define a new orientation for urban SDM. SDM has historically excelled at forecasting where land-use change is likely to occur, but it has been less successful in informing how cities can act to achieve sustainability goals. The challenge before the field is to reposition itself from being a predictive exercise to becoming a platform for navigating urban transitions.

At the heart of this repositioning is a redefinition of what counts as success. So far, most SDM studies continue to evaluate performance by map accuracy alone. While predictive validity remains valuable, it is no longer sufficient. Cities facing climate neutrality targets, rising inequality, and systemic risks need models that provide actionable insights into trade-offs and interventions. SDM therefore needs to broaden its evaluative criteria. The quality of a model ought to be assessed by whether it reduces vulnerability to climate hazards, enlarges equitable accessibility, and guides decision makers toward strategies that are robust under uncertainty.

A second element of repositioning concerns actionability. Policymakers operate with specific levers such as zoning reforms, infrastructure investments, or environmental regulations. Too often, SDM outputs do not translate into this decision space. By embedding accessibility metrics and resilience indicators, models can begin to generate outputs that align directly with the questions governments face. For example, rather than presenting a probability surface of sprawl, a model could show which zoning options most effectively expand access to jobs within feasible commuting times. Such outputs strengthen the link between SDM and governance and build trust among stakeholders. In this sense, the future of SDM aligns with insights from the PSS tradition: The aim is not to prescribe solutions, but to provide structured support for evaluating alternatives (Geertman and Stillwell 2020).

The third element is inclusivity and scalability. Current practice continues to be concentrated in a narrow set of data-rich cities, yet sustainability transitions are a global imperative. The future of SDM should therefore prioritize methods that work across diverse geographies, including small, medium, and shrinking cities. Lightweight, transferable cores, enriched by open semantic layers, could allow any municipality to run a baseline model. Modular extensions would provide opportunities for richer analysis where resources permit. This approach ensures that SDM is not the preserve of well-funded megacities, but a common resource available to all.

The fourth element is a resilience orientation. Contemporary urban systems are increasingly defined by shocks. A planning tool that fails to capture thresholds, cascading failures, or network vulnerabilities risks producing scenarios that are reassuring but misleading. Incorporating network science and space syntax into SDM provides a way to embed resilience directly into scenario testing (Hillier and Hanson 1989; De Domenico et al. 2015). This allows cities to ask, for example, how robust their accessibility would remain under a flood event, or how service consolidation during economic downturns would affect vulnerable populations. By placing resilience at the center, SDM becomes a partner in preparing cities for uncertainty rather than simply extrapolating from the past.

Finally, SDM needs to position itself within the emerging landscape of urban digital infrastructures. Many municipalities are investing in digital twins—platforms that integrate real-time data, visualization, and simulation. Although still experimental, these systems present a clear opportunity for SDM to become operational in planning practice. By providing hybrid models, semantic layers, and resilience metrics, SDM can complement digital twins with structured, theory-informed scenario analysis. This integration would require governance safeguards around transparency, data privacy, and inclusivity, but it represents a powerful avenue for embedding SDM directly in decision-making workflows.

### A research agenda

The repositioning of SDM requires a concerted research agenda over the coming decade. In the short term, the most urgent task is to develop hybrid AI–theory prototypes and test them across diverse city types. Demonstrating that these models can improve predictive performance while remaining interpretable will be essential for broader adoption. A second short-term priority is the creation of open semantic urban layers derived from foundation models such as vision-language architectures. Equally pivotal is advancing methods to integrate the space–time prism into SDM as a standard accessibility evaluation tool. Doing so would allow researchers and practitioners to compare how alternative futures affect the feasible activity spaces of different population groups, thereby operationalizing equity concerns in urban transitions. Publicly available datasets of urban functions, with transparent documentation and validation, would significantly reduce the entry barriers for cities with limited data. A third priority is the development of shrinkage-aware models, explicitly simulating service consolidation and network rightsizing. So far, such models continue to be rare, despite the growing number of shrinking cities worldwide.

In the medium term, the research agenda ought to focus on evaluation benchmarks. The climate science community has long benefitted from shared datasets and coordinated assessments. SDM could establish similar benchmarks that assess not only map accuracy but also accessibility equity, resilience under stress, and cross-context transferability. Standardized evaluation would help the field accumulate cumulative knowledge rather than fragmented case studies. Another medium-term priority is the expansion of policy experiments. Collaborations between researchers and municipalities could integrate SDM into real planning decisions, providing feedback on usability and legitimacy. Participatory modeling approaches, in which stakeholders co-design scenarios, can further enhance the practical impact of SDM.

In the long term, the vision is for SDM to become a co-production platform. Rather than a tool used solely by modelers, SDM could evolve into a space where scientists, policymakers, and communities jointly explore futures. Hybrid AI–theory architectures would provide analytical strength, space–time accessibility would ensure human relevance, network-based metrics would secure resilience, semantic functions would enrich context, and scalable frameworks would guarantee inclusivity. Together, these elements would create not a monolithic forecasting engine but a flexible, interpretable partner for urban transitions.

What deserves emphasis, however, are the blind spots that the field has yet to confront. Urban futures are deeply uncertain, and any attempt to present them as deterministic will inevitably disappoint. Robustness under multiple plausible futures is therefore as pivotal as accuracy against the past. Equally, urban change is inseparable from environmental processes including water, heat, biodiversity, and carbon, and SDM will continue to be partial until it is coupled to these dynamics. Finally, the adoption of foundation models forces us to ask uncomfortable but necessary questions about bias, transparency, and data justice. Addressing these challenges may not yield elegant models, but it will yield models that cities can trust. That, in the end, may prove to be the most decisive measure of SDM’s success.

### A policy agenda

Alongside scientific research, a parallel policy agenda is essential. Municipalities can begin by adopting minimum standards for SDM deployment. These might include open documentation of assumptions, reporting of uncertainty, inclusion of accessibility and resilience metrics, and participatory validation with stakeholders. Such standards would increase transparency and trust in the modeling process.

International organizations and funding bodies can support the development of open-source SDM platforms. By investing in shared infrastructure and tools, they can reduce duplication of effort and ensure that resource-constrained cities have access to usable modeling technology. Policies should also prioritize capacity building—training planners and local officials in interpreting and applying SDM outputs. Without such capacity, even the most advanced models will continue to be underutilized. Building human capital and institutional knowledge addresses the implementation gap from the demand side, enabling the complex models to be effectively translated into planning practice.

Finally, global collaboration is vital. Just as climate science has advanced through coordinated modeling frameworks and intercomparison projects, SDM could benefit from international networks that pool data, share methods, and develop collective assessments. Such collaboration would accelerate innovation and ensure that progress is distributed equitably, rather than concentrated in a handful of leading institutions. A global SDM community, supported by international bodies, could take on benchmarking exercises and cross-city studies that no single team could accomplish, thereby pushing the field forward more rapidly.

Policies must also anticipate the new responsibilities that advanced modeling will bring. If models are to grapple with uncertainty, governments must be prepared to act on ranges of possible outcomes, not just single-point predictions. If SDM is to be coupled with environmental processes, policy frameworks must be ready to confront trade-offs between growth, ecological limits, and climate commitments. And if foundation models are to shape the semantic layers of urban knowledge, then principles of openness, fairness, and accountability cannot be an afterthought—they need to be designed into institutions from the start. The future of SDM will be written not only in code; it will be written in the governance frameworks that determine who has access, who bears responsibility, and whose futures are made visible.

## Conclusion

Urban SDM has matured into a technically sophisticated field, yet it now stands at a crossroads. For more than three decades, it has provided valuable insights into patterns of urban growth and land-use change. But the sustainability challenges of the twenty-first century demand more than accurate forecasts. Cities today need tools that help them navigate transitions toward climate neutrality, resilience, and equity.

In this perspective, we identified the key limitations of current SDM practice that undermine its contribution to sustainability transitions, and we argued that the field must reposition itself from a purely predictive device to a transition-support platform. To achieve this, we outlined five interlinked pathways that together reposition SDM from pattern-focused forecasting to a transition-support platform aligned with climate neutrality, resilience, and equity. Together, these five pathways directly address the specific shortcomings elaborated in “Limitations of current SDM practice” section illustrating how SDM can evolve in a coherent and purposeful manner rather than through isolated methodological advances.

This repositioning creates a more integrative SDM architecture: one that moves beyond reproducing past patterns, supports actionable planning decisions, and remains usable across diverse urban contexts, including cities undergoing silent urbanization or shrinkage. Their real strength lies in combination: shifting SDM from reproducing past patterns to enabling strategies for sustainable and just urban futures.

Looking ahead, a broader agenda comes into focus. SDM should be evaluated not only by predictive accuracy but also by its ability to expand equitable accessibility, preserve resilience under stress, and align outputs with governance choices. Achieving this shift will require collective investment in open semantic datasets, shared evaluation benchmarks, and training programs that build local capacity. Just as climate science has advanced through common infrastructures and coordinated assessments, SDM can benefit from collaborative platforms that accelerate innovation while ensuring equitable distribution of benefits. Participatory and transparent approaches will be decisive in securing legitimacy for these models in real-world planning processes.

Ultimately, the strength of SDM will be measured not only by what it predicts but also by how effectively it supports cities navigating uncertainty and designing fairer, more resilient futures. The choice facing the field is clear. SDM can remain focused on reproducing the past, or it can embrace a broader mission as a co-production platform where scientists, policymakers, and communities jointly explore pathways for sustainable urban transitions. If the latter path is pursued, SDM has the potential to become a cornerstone of global sustainability science—helping cities not only to anticipate change but also to actively shape it. The opportunity is urgent, and the responsibility profound.

## References

[CR1] Anestis, G., and D. Stathakis. 2024. Urbanization trends from global to the local scale. In *Geographical Information Science*, pp. 357–375. Amsterdam: Elsevier. 10.1016/B978-0-443-13605-4.00010-2.

[CR2] Bai, X. 2025. Cities as transformative agents for global sustainability. *Science* 389: 3642. 10.1126/science.adz3642. 40638740

[CR3] Batty, M. 2013. *The new science of cities*. Cambridge, MA: MIT Press.

[CR4] Batty, M. 2018. Digital twins. *Environment and Planning b: Urban Analytics and City Science* 45: 817–820. 10.1177/2399808318796416.

[CR5] Beilin, R., and C. Wilkinson. 2015. Introduction: Governing for urban resilience. *Urban Studies* 52: 1205–1217. 10.1177/0042098015574955.

[CR6] Bettencourt, L.M.A. 2013. The origins of scaling in cities. *Science* 340: 1438–1441. 10.1126/science.1235823. 23788793

[CR7] Bettencourt, L.M.A., J. Lobo, D. Helbing, C. Kühnert, and G.B. West. 2007. Growth, innovation, scaling, and the pace of life in cities. *Proceedings of the National Academy of Sciences* 104: 7301–7306. 10.1073/pnas.0610172104. PMC1852329 17438298

[CR8] Bixler, R.P., M. Coudert, S.M. Richter, J.M. Jones, C. Llanes Pulido, N. Akhavan, M. Bartos, P. Passalacqua, et al. 2022. Reflexive co-production for urban resilience: Guiding framework and experiences from Austin, Texas. *Frontiers in Sustainable Cities* 4: 1015630. 10.3389/frsc.2022.1015630.

[CR9] Boutreux, T., A. Ezcurra, A. Lemée, A. Podevyn, C. Bréteau, and G. Bonnin. 2024. Addressing the sustainable urbanism paradox: Tipping points in morphology and greening. *NPJ Urban Sustainability* 4: 38. 10.1038/s42949-024-00176-7.

[CR10] Cai, Z. 2025. Evolving from rules to learning in urban modeling and planning support systems. *Urban Science* 9: 508. 10.3390/urbansci9120508.

[CR11] Cai, Z., B. Wang, C. Cong, and V. Cvetkovic. 2020. Spatial dynamic modelling for urban scenario planning: A case study of Nanjing, China. *Environment and Planning b: Urban Analytics and City Science* 47: 1380–1396. 10.1177/2399808320934818.

[CR12] Cai, Z., Y. Kwak, V. Cvetkovic, B. Deal, and U. Mörtberg. 2023. Urban spatial dynamic modeling based on urban amenity data to inform smart city planning. *Anthropocene* 42: 100387. 10.1016/j.ancene.2023.100387.

[CR62] Cai, Z., A. Karvonen, C. Cong, and W. Huang. 2026. Fine-grained urban land use simulation: Integrating spatial dynamic modeling with a pre-trained vision-language model. *Computers Environment and Urban Systems* 126: 102416. 10.1016/j.compenvurbsys.2026.102416

[CR64] Carson, D.A., D.B. Carson, and N. Argent. 2022. Cities hinterlands and disconnected urban-rural development: Perspectives from sparsely populated areas. *Journal of Rural Studies* 93: 104–111. 10.1016/j.jrurstud.2022.05.012

[CR13] Chakraborty, T.C., X. Li, G. Wang, C. Sarangi, K. Schumacher, A. Ganju, J. Rao, and G. Ban-Weiss. 2024. Large disagreements in estimates of urban land across scales and their implications. *Nature Communications* 15: 9165. 10.1038/s41467-024-52241-5. PMC11502887 39448573

[CR14] Clarke, K.C., S. Hoppen, and L. Gaydos. 1997. A self-modifying cellular automaton model of historical urbanization in the San Francisco Bay area. *Environment and Planning b: Planning and Design* 24: 247–261. 10.1068/b240247.

[CR15] De Domenico, M., A. Sole-Ribalta, E. Omodei, S. Gómez, and A. Arenas. 2015. Ranking in interconnected multilayer networks reveals versatile nodes. *Nature Communications* 6: 6868. 10.1038/ncomms7868. 25904405

[CR16] De Domenico, M., C. Granell, M.A. Porter, and A. Arenas. 2016. The physics of spreading processes in multilayer networks. *Nature Physics* 12: 901–906. 10.1038/nphys3865.

[CR17] Dosovitskiy, A., L. Beyer, A. Kolesnikov, D. Weissenborn, X. Zhai, T. Unterthiner, M. Dehghani, M. Minderer, et al. 2021. An image is worth 16×16 words: Transformers for image recognition at scale. In *International Conference on Learning Representations (ICLR)*.

[CR18] Evans, J., A. Karvonen, and R. Raven. 2016. The experimental city: New modes and prospects of urban transformation. In *The experimental city*, ed. J. Evans, A. Karvonen, and R. Raven, 1–12. London: Routledge.

[CR19] Filatova, T., P.H. Verburg, D.C. Parker, and C.A. Stannard. 2013. Spatial agent-based models for socio-ecological systems: Challenges and prospects. *Environmental Modelling & Software* 45: 1–7. 10.1016/j.envsoft.2013.03.017.

[CR20] Gao, J., B. Barzel, and A.-L. Barabási. 2016. Universal resilience patterns in complex networks. *Nature* 530: 307–312. 10.1038/nature16948. 26887493

[CR61] Gao, S., H. Jansen, and B.D. Ryan. 2023. Demolition after decline: Understanding and explaining demolition patterns in US and German shrinking cities. *Cities* 134: 104185. 10.1016/j.cities.2022.104185

[CR21] Geertman, S. 2006. Potentials for planning support: A planning-conceptual approach. *Environment and Planning b: Planning and Design* 33: 863–880. 10.1068/b31129.

[CR22] Geertman, S. 2016. PSS: Beyond the implementation gap. *Transportation Research Part a: Policy and Practice* 104: 70–76. 10.1016/j.tra.2016.10.016.

[CR23] Geertman, S., and J. Stillwell, eds. 2020. *Handbook of planning support science*. Cheltenham: Edward Elgar.

[CR24] Geurs, K. T., and B. van Wee. 2004. Accessibility evaluation of land-use and transport strategies: Review and research directions. *Journal of Transport Geography* 12: 127–140. 10.1016/j.jtrangeo.2003.10.005.

[CR25] Haase, A., M. Bernt, K. Großmann, V. Mykhnenko, and D. Rink. 2016. Varieties of shrinkage in European cities. *European Urban and Regional Studies* 23: 86–102. 10.1177/0969776413481985.

[CR26] Hägerstrand, T. 1968. *Innovation diffusion as a spatial process*. Chicago: University of Chicago Press.

[CR27] Hillier, B., and J. Hanson. 1989. *The social logic of space*. Cambridge: Cambridge University Press.

[CR28] Huang, W., J. Wang, and G. Cong. 2024. Zero-shot urban function inference with street view images through prompting a pretrained vision-language model. *International Journal of Geographical Information Science* 38: 1414–1442. 10.1080/13658816.2024.2347322.

[CR29] IEA. 2021. *World Energy Outlook 2021*. Paris: International Energy Agency.

[CR30] Intergovernmental Panel on Climate Change (IPCC). 2023. *Climate Change 2022: Impacts, Adaptation and Vulnerability. Contribution of Working Group II to the Sixth Assessment Report of the Intergovernmental Panel on Climate Change*. Cambridge: Cambridge University Press.

[CR31] Johnson, C., E. Osuteye, T. Ndezi, and F. Makoba. 2022. Co-producing knowledge to address disaster risks in informal settlements in Dar es Salaam, Tanzania: Pathways toward urban equality? *Environment and Urbanization* 34: 349–371. 10.1177/09562478221112256.

[CR32] Karvonen, A., V. Cvetkovic, P. Herman, K. Johansson, H. Kjellström, M. Molinari, and M. Skoglund. 2021. The ‘New Urban Science’: Towards the interdisciplinary and transdisciplinary pursuit of sustainable transformations. *Urban Transformations* 3: 9. 10.1186/s42854-021-00028-y.

[CR33] Kivelá, M., A. Arenas, M. Barthelemy, J.P. Gleeson, Y. Moreno, and M.A. Porter. 2014. Multilayer networks. *Journal of Complex Networks* 2: 203–271.

[CR34] Li, X., and P. Gong. 2016. Urban growth models: Progress and perspective. *Science Bulletin* 61: 1637–1650. 10.1007/s11434-016-1111-1.

[CR35] Liu, X., X. Liang, X. Li, X. Xu, J. Ou, Y. Chen, Y. Li, and L. Jiang. 2017. A future land-use simulation model (FLUS) for simulating multiple land-use scenarios by coupling human and natural effects. *Landscape and Urban Planning* 168: 94–116. 10.1016/j.landurbplan.2017.09.019.

[CR36] Liu, H., R. Homma, Q. Liu, and C. Fang. 2021. Multi-scenario prediction of intra-urban land use change using a cellular automata-random forest model. *ISPRS International Journal of Geo-Information* 10: 503. 10.3390/ijgi10080503.

[CR37] Liu, F., D. Chen, Z. Guan, X. Zhou, J. Zhu, Q. Ye, L. Fu, and J. Zhou. 2024. RemoteCLIP: A vision language foundation model for remote sensing. *IEEE Transactions on Geoscience and Remote Sensing* 62: 1–14. 10.1109/TGRS.2024.3390838.

[CR38] Lu, S., J. Guo, J.R. Zimmer-Dauphinee, J.M. Nieusma, X. Wang, P. VanValkenburgh, S.A. Wernke, and Y. Huo. 2025. Vision foundation models in remote sensing: A survey. *IEEE Geoscience & Remote Sensing Magazine *13(3): 190–215. 10.1109/MGRS.2025.3541952.

[CR39] Martínez-Fernández, C., I. Audirac, S. Fol, and E. Cunningham-Sabot. 2012. Shrinking cities: Urban challenges of globalization. *International Journal of Urban and Regional Research* 36: 213–225. 10.1111/j.1468-2427.2011.01092.x. 22518881

[CR40] McGranahan, G. 2015. Realizing the right to sanitation in deprived urban communities: Meeting the challenges of collective action, coproduction, affordability, and housing tenure. *World Development* 68: 242–253. 10.1016/j.worlddev.2014.12.008.

[CR41] Meerow, S., and J.P. Newell. 2019. Urban resilience for whom, what, when, where, and why? *Urban Geography* 40: 309–329. 10.1080/02723638.2016.1206395.

[CR42] Neutens, T., T. Schwanen, and F. Witlox. 2011. The prism of everyday life: Towards a new research agenda for time geography. *Transport Reviews* 31: 25–47. 10.1080/01441647.2010.484153.

[CR43] Parker, D.C., S.M. Manson, M.A. Janssen, M.J. Hoffmann, and P. Deadman. 2003. Multi-agent systems for the simulation of land-use and land-cover change: A review. *Annals of the Association of American Geographers* 93: 314–337. 10.1111/1467-8306.9302004.

[CR44] Penn, A. 2003. Space syntax and spatial cognition: Or, why the axial line? *Environment and Behavior* 35: 30–65. 10.1177/0013916502238864.

[CR45] Peponis, J., S. Bafna, and Z. Zhang. 2008. The connectivity of streets: Reach and directional distance. *Environment and Planning b: Planning and Design* 35: 881–901. 10.1068/b33088.

[CR46] Pereira, R.H.M., T. Schwanen, and D. Banister. 2017. Distributive justice and equity in transportation. *Transport Reviews* 37: 170–191. 10.1080/01441647.2016.1257660.

[CR47] Pickett, S.T.A., N. Frantzeskaki, E. Andersson, A.S. Barau, D.L. Childers, F.-A. Hoover, A.E. Lugo, T. McPhearson, et al. 2024. Shifting forward: Urban ecology in perspective. *Ambio* 53: 890–897. 10.1007/s13280-024-02007-6. PMC11058125 38642313

[CR48] Radford, A., J. Kim, C. Hallacy, A. Ramesh, G. Goh, S. Agarwal, G. Sastry, A. Askell, et al. 2021. Learning transferable visual models from natural language supervision. In *Proceedings of the 38th International Conference on Machine Learning (ICML)* pp. 8748–8763.

[CR49] Rudin, C. 2019. Stop explaining black box machine learning models for high stakes decisions and use interpretable models instead. *Nature Machine Intelligence* 1: 206–215. 10.1038/s42256-019-0048-x. PMC9122117 35603010

[CR50] Schindler, S. 2017. Towards a paradigm of Southern urbanism. *City* 21: 47–64. 10.1080/13604813.2016.1263494.

[CR51] Seto, K.C., B. Güneralp, and L.R. Hutyra. 2012. Global forecasts of urban expansion to 2030 and direct impacts on biodiversity and carbon pools. *Proceedings of the National Academy of Sciences* 109: 16083–16088. 10.1073/pnas.1211658109. PMC3479537 22988086

[CR52] Sharifi, A., and Y. Yamagata. 2018. Resilience-oriented urban planning. In *Resilience-oriented urban planning: Theoretical and empirical insights*, pp. 3–27. Cham: Springer International Publishing. 10.1007/978-3-319-75798-8_1.

[CR53] Steffen, W., Å. Persson, L. Deutsch, J. Zalasiewicz, M. Williams, K. Richardson, C. Crumley, P. Crutzen, et al. 2011. The Anthropocene: From global change to planetary stewardship. *Ambio* 40: 739–761. 10.1007/s13280-011-0185-x. PMC3357752 22338713

[CR54] UN-Habitat. 2020. *World Cities Report 2020: The Value of Sustainable Urbanization.* Nairobi: United Nations Human Settlements Programme (UN-Habitat).

[CR55] Verburg, P.H., P. Alexander, T. Evans, N.R. Magliocca, Z. Malek, M.D.A. Rounsevell, and J. van Vliet. 2019. Beyond land cover change: Towards a new generation of land use models. *Current Opinion in Environmental Sustainability* 38: 77–85. 10.1016/j.cosust.2019.05.002.

[CR56] Voinov, A., and F. Bousquet. 2010. Modelling with stakeholders. *Environmental Modelling & Software* 25: 1268–1281. 10.1016/j.envsoft.2010.03.007.

[CR57] White, R., and G. Engelen. 1993. Cellular automata and fractal urban form: A cellular modelling approach to the evolution of urban land-use patterns. *Environment and Planning a: Economy and Space* 25: 1175–1199. 10.1068/a251175.

[CR58] Williams, D.S., M. Máñez Costa, C. Sutherland, L. Celliers, and J. Scheffran. 2019. Vulnerability of informal settlements in the context of rapid urbanization and climate change. *Environment and Urbanization* 31: 157–176. 10.1177/0956247818819694.

[CR59] Wu, N., and E.A. Silva. 2010. Artificial intelligence solutions for urban land dynamics: A review. *Journal of Planning Literature* 24: 246–265. 10.1177/0885412210361571.

[CR63] Zhang, H., and Z. Cai. 2026. Advancing hybrid spatial dynamic modeling for urban sustainable transitions. *Discover Cities* 3: 34. 10.1007/s44327-026-00220-4

[CR60] Zhou, Y., X. Li, W. Chen, L. Meng, Q. Wu, P. Gong, and K.C. Seto. 2022. Satellite mapping of urban built-up heights reveals extreme infrastructure gaps and inequalities in the Global South. *Proceedings of the National Academy of Sciences* 119(46). 10.1073/pnas.2214813119 PMC9674218 36343227

